# P-103. Adenoviremia Clearance Following Treatment with Intravenous (IV) Brincidofovir (BCV) in Immunocompromised Patients is Associated with Positive Clinical Disease Response: Preliminary Outcomes from the ATHENA Phase IIa Study

**DOI:** 10.1093/ofid/ofae631.310

**Published:** 2025-01-29

**Authors:** Gabriela Maron, Michael J Boeckh, Genovefa Papanicolaou, Carlos Gomez, Vinod Prasad, Jasmeen Dara, Caitlin Elgarten, Madan Kumar, Robert Wynn, Thomas Lion, Koji Fukushima, Rochelle Maher, Nkechi Azie, Michael Grimley

**Affiliations:** St. Jude Children's Research Hospital, Memphis, Tennessee; Fred Hutchinson Cancer Center, Seattle, WA; Memorial Sloan Kettering Cancer Center, New York, NY; University of Nebraska Medical Center, Omaha, Nebraska; Duke Children's Hospital and Health Center, Durham, North Carolina; University of California, San Francisco, California; Childrens Hospital of Philadelphia, Philadelphia, Pennsylvania; University of Chicago Medicine, Chicago, Illinois; Royal Manchester Children's Hospital, Manchester, England, United Kingdom; St. Anna Children's Cancer Research Institute, Vienna, Wien, Austria; SymBio Pharmaceuticals Limited, Minato, Tokyo, Japan; SymBio Pharma USA, Inc., Durham, North Carolina; SymBio Pharma USA, Inc., Durham, North Carolina; University of Cincinnati College of Medicine, Cincinnati, Ohio

## Abstract

**Background:**

Adenovirus (AdV) may cause fatal infections in immunocompromised patients. There are no drugs approved for treatment of serious AdV infections. BCV, a lipid conjugate of cidofovir, antiviral with enhanced ADV activity without renal or haematological toxicity. The value of viremia clearance in predicting clinical response has been a source of scientific debate. Athena is a multiple ascending dose study of the safety and efficacy of IV BCV in treatment of serious AdV infection. NCT04706923.

**Figure:**
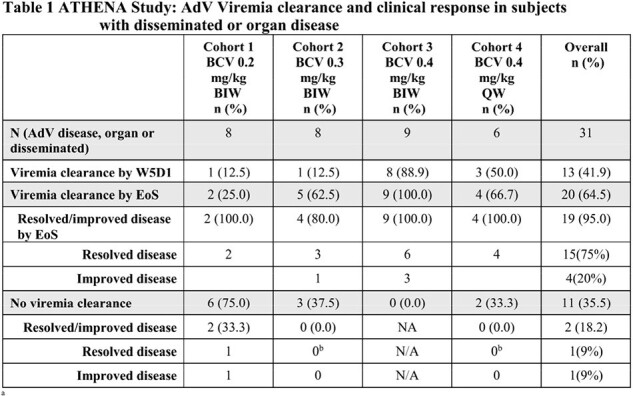

**Methods:**

Eligible immunocompromised patients aged ≥ 2 months with adenoviremia were treated with IV BCV for 4 to 14 weeks (until viremia clearance). The dosing regimen was 0.2; 0.3; 0.4 mg/kg twice weekly (BIW) in Cohorts 1;2;3 respectively, or 0.4 mg/kg weekly (Cohort 4). For all cohorts the dose was capped at the 50kg equivalent. ADV-disease was defined by organ system involvement as localized (1 organ system) or disseminated ( >1 organ system or fever and elevated serum aminotransferases). All nine patients with baseline disease in Cohort 3 (0.4mg/kg BIW) achieved viremia clearance, 89% within 4 weeks. Clearance of adenoviremia was monitored by weekly quantitative PCR. Clinical response in terms of resolution or improvement of AdV disease as assessed by treating physicians is summarized here.

**Results:**

Thirty-four patients were enrolled and treated. ADV-viremia response was dose-dependent. The majority 31/34 (91%) of patients had AdV disease at baseline (Table 1). Among the 20/31 patients who achieved viral clearance,19/20 (95%) showed a positive clinical response with resolution (75%) or improvement of the AdV disease(20%). Among the patients who failed to achieve viral clearance 2/11 (18%) showed resolution(9%) or improvement(9%) of the AdV disease.

**Conclusion:**

IV BCV therapy cleared ADV viremia in a dose-dependent manner. ADV-viremia clearance was associated with positive clinical response rate. These results support the potential utility of IV BCV for the treatment of AdV infection and viremia clearance as a surrogate marker of clinical response.

**Disclosures:**

**Gabriela Maron, MD, MS**, Astellas Inc: Grant/Research Support|NIH: Grant/Research Support|SymBio Pharma: Advisor/Consultant|SymBio Pharma: Grant/Research Support **Michael J. Boeckh, MD PhD**, Allovir: Advisor/Consultant|Allovir: Grant/Research Support|AstraZeneca: Advisor/Consultant|AstraZeneca: Grant/Research Support|Merck: Advisor/Consultant|Merck: Grant/Research Support|Moderna: Advisor/Consultant|Moderna: Grant/Research Support|Symbio: Advisor/Consultant **Genovefa Papanicolaou, MD**, AlloVir: Advisor/Consultant|AlloVir: Data safety monitoring committee|Merck: Advisor/Consultant|Merck: Grant/Research Support|Merck: Investigator|Symbio: Advisor/Consultant **Robert Wynn, MD, MRCP, FRCPath**, AVRO BIO: Grant/Research Support|AVRO BIO: Milestone payments MPSII clinical trial|Orchard Therapeutics: Grant/Research Support|Orchard Therapeutics: Milestone payments MPSII clinical trial **Thomas Lion, MD, PhD**, SymBio Pharmaceuticals Limited: Advisor/Consultant|SymBio Pharmaceuticals Limited: Grant/Research Support **Koji Fukushima, MD, PhD**, SymBio Pharmaceuticals Limited: Employee|SymBio Pharmaceuticals Limited: Stocks/Bonds (Private Company) **Rochelle Maher, MS**, SymBio Pharma USA: Employee **Nkechi Azie, MD MBA FIDSA**, SymBio Pharma USA: Employee **Michael Grimley, MD**, SymBio Pharmaceuticals Limited: Advisor/Consultant

